# Maintenance of translational elongation rate underlies the survival of *Escherichia coli* during oxidative stress

**DOI:** 10.1093/nar/gkz467

**Published:** 2019-05-27

**Authors:** Manlu Zhu, Xiongfeng Dai

**Affiliations:** School of Life Sciences, Central China Normal University, Wuhan, Hubei province, China

## Abstract

To cope with harsh circumstances, bacterial cells must initiate cellular stress response programs, which demands the *de novo* synthesis of many stress defense proteins. Reactive oxygen species (ROS) is a universal environmental stressor for both prokaryotic cells and eukaryotic cells. However, the physiological burden that limits the survival of bacterial cells during oxidative stress remains elusive. Here we quantitatively characterize the cell growth and translational elongation rate of *Escherichia coli* cells treated with different doses of hydrogen peroxide. Cell growth is immediately arrested by low to moderate levels of hydrogen peroxide, but completely recovers after a certain lag time. The lag time depends positively on the dose of hydrogen peroxide. During the lag time, translational elongation rate drops by as much as ∼90% at initial stage and recovers to its normal state later, a phenomenon resulting from the dramatic alteration in cellular tRNA pools during oxidative stress. However, translational elongation is completely stalled at a certain threshold-level of hydrogen peroxide, at which cells ultimately fail to resume growth. Although the mRNA transcription of oxidative defense genes in *oxyR* regulon is dramatically induced upon hydrogen peroxide treatment, the extreme slow-down of translational elongation during high levels of hydrogen peroxide has severely compromised the timely synthesis of those oxidative defense proteins. Our study demonstrates that the tRNA-limited translational elongation is a key physiological bottleneck that the bacteria must overcome to counteract ROS, and the maintenance of translational elongation rate for timely synthesis of stress defense proteins is crucial for cells to smoothly get over the oxidative stress.

## INTRODUCTION

In nature, bacterial cells frequently undergo many harsh environmental conditions such as nutrient deprivation, oxidants, heat, low pH and high osmolarity, which inhibit the growth and survival of bacteria cells. To maintain viability and ultimately overcome stress conditions, bacterial cells must initiate certain stress response programs, triggering the *de novo* synthesis of many stress-defense proteins (1). One of best-characterized example is the *rpoS*-regulon (2,3), which contains a list of over 100 genes encoding a diverse set of proteins functioning in stress management, DNA repair, central metabolism and cell morphology control (1,2,4). During nutrient starvation and other stress conditions, the expression of genes in *rpoS*-regulon is strongly induced, protecting bacteria against the potential damage of external stress (4–6). Failure in timely synthesis of those stress-defense proteins could cause significant loss in cell viability (4,7).

Reactive oxygen species (ROS), including hydrogen peroxide (H_2_O_2_), superoxide anions (O_2_^−^) and hydroxyl radical (•OH), is a universal environmental stressor for almost all types of cells including bacterial cells, yeast cells and mammalian cells (8–10). For human beings, it has long been proposed that ROS-induced oxidative stress is strongly implicated in the emergence of many important diseases and disorders such as aging/senescence, cancer, cardiovascular diseases, neurodegenerative disorder, rheumatoid arthritis and inflammation (8,9,11). Severe oxidative stress causes damage of intracellular macromolecules including proteins, nucleic acids and lipids (8,9,12). When confronting oxidative stress, bacterial cells, yeast cells as well as mammalian cells undergo immediate growth arrest due to inhibition of the activities of certain key metabolic enzymes (13–16). Cells then must initiate specific ROS-defense signaling pathways to remove intracellular ROS and resume growth (8,14,16,17). The cellular response to oxidative stress in the model bacteria species has been largely elucidated. For *Escherichia coli*, the *oxyR* regulon (primarily responds to H_2_O_2_) and *soxRS* regulon (primarily responds to O_2_^−^) defend cells against the damage of ROS (16–19). When *E. coli* cells are treated with a low dose of H_2_O_2_, growth arrest occurs immediately and the expression of ∼30 genes is maximally induced within 10–30 min (18,20–22). Among them, the oxidized form of the transcriptional regulator, OxyR, induces a dozen of genes such as *katG* (encoding catalase G), *ahpCF* (encoding alkyl hydroperoxide reductase), *trxC* (encoding reduced thioredoxin 2),to remove intracellular H_2_O_2_, maintain redox homeostasis and ultimately enable cells to resume growth (16,17,22). The *oxyR* mutant strain, which fails to induce related defense proteins, becomes hypersensitive to H_2_O_2_ shock (18,23,24). However, even for wild type cells, it cannot survive and resume growth when the external H_2_O_2_ level becomes too high. Therefore, some fundamental questions remain open: what's the major physiological burden that limits the survival of bacteria during oxidative stress? What's the major factor that determines whether bacteria could smoothly survive and ultimately get over the oxidative stress?

Under stress conditions, cell growth and the overall protein synthesis are severely inhibited (5,6,25,26). The overall protein synthesis rate is limited by the number of actively translating ribosomes and the translational elongation rate (alternatively, polypeptide chain elongation rate) (25–27). When *E. coli* cells grow in rich nutrient conditions, the ribosome translates proteins at a high elongation rate (16–17 amino acids per sec, aa/s) (25,27–29). When growth is arrested during nutrient deprivation (e.g. carbon, nitrogen and amino acid), the overall protein synthesis rate is severely inhibited. However, *E. coli* cells still maintain a moderate translational elongation rate (8-9 aa/s) so that it can timely synthesize stress-related proteins to survive in those extreme poor conditions (25,27,30,31). In this study, we quantitatively characterize the cell growth and translational elongation rate of *E. coli* being subjected to hydrogen peroxide (H_2_O_2_) treatment. We find that oxidative stress causes unusually dramatic slow-down or even complete stalling of the translational elongation in *E. coli* through substantially down-regulating cellular tRNA pools. The tRNA-limited translational elongation process, being crucial for the timely synthesis of stress defense proteins, becomes a key physiological bottleneck that limits the survival of *E. coli* cells during oxidative stress.

## MATERIALS AND METHODS

### Strain and medium

Bacterial strains used in this study were wild type K-12 *E. coli* NCM3722 strain (32,33) and its derivatives NQ1468 (25), FL174, FL175, FL189, FL190, FL191 and FL192 strains. The NQ1468 strain was used in LacZα induction assay for calibration of initiation time cost in the translational elongation rate measurement. FL174 and FL175 strain were used for measuring the translational elongation rate of ManA-GFP and FusA-GFP protein, respectively. The FL189 strain was used for Rnase D overexpression experiment. The FL190, FL191 and FL192 strains are strains harboring OxyR-regulated translational-fused GFP proteins.

The FL174 strain and FL175 strain harbored pFL-manA-gfp vector and pFL-fusA-gfp vector, respectively. To make these two vectors, a *Placq-lacI* cassette together with its downstream *Ptac* promoter (without RBS) were first inserted into the XhoI/EcoRI sites of the pZE11 vector; a pair of NdeI/BamHI sites was introduced downstream of *Ptac* promoter through point mutation. The *manA* and *fusA* gene of *E. coli* were then PCR amplified and inserted into the NdeI/BamHI sites. Finally, the coding sequence of *egfp*, together with a N-terminal 30 bp sequence encoding (GGGGS)_2_ linker was placed downstream of the *manA* and *fusA*, respectively, yielding pFL-manA-gfp and pFL-fusA-gfp vector, respectively. The pFL-manA-gfp and pFL-fusA-gfp were then transformed into *E. coli*, generating FL174 and FL175 strain for measuring translational elongation rates of GFP fusion proteins.

To construct three strains (FL190, FL191 and FL192) harboring OxyR-regulated translational-fused GFP proteins, the ORFs of *dps, trxC* and *grxA* genes together with their upstream ∼200 bp transcriptional regulator regions were PCR amplified and inserted into the XhoI/BamHI site of the pFL-manA-gfp vector to replace the whole *Placq-lacI-Ptac-manA* cassette. In the case, the three translational-fused GFP proteins were controlled by the native OxyR-regulated promoters. The three vectors were then transformed into NCM3722 strain to obtain FL190, Fl191 and FL192 strain, respectively. These three strains were used for the experiments of Figure 5H–J.

To construct an Rnase D-overexpression strain, FL189, the coding sequence of *rnd* gene was PCR amplified using T5 direct PCR kit (Tsingke Biotech) and first inserted into pClone007 Blunt vector (Tsingke Biotech) for sequence verification. The sequence-verified *rnd* fragment was then inserted into the NdeI/SpeI of pFL-manA-gfp vector to replace the *manA-gfp* gene so that *rnd* expression was controlled by *Ptac* promoter, yielding pFL-rnd vector. The pFL-rnd vector was further transformed into NCM3722 strain for Rnase D overexpression experiments in Figure 6.

MOPS-buffered minimal medium was the same as used in Cayley *et al.* (34) using 0.2% glucose as the carbon source and 10 mM NH_4_Cl as the nitrogen source. The MOPS reagent was ordered from Coolaber Biotech in Beijing.

### Cell growth

Cell growth experiments were performed in a water bath shaker (220 rpm under 37°C). A standard procedure of cell growth experiments included three steps: seed culture, pre-culture and experimental culture. Cells from a fresh colony were inoculated into LB medium (Coolaber Biotech) and grew for several hours as seed culture. Seed culture was inoculated into MOPS glucose minimal medium for growing overnight as pre-culture. At the next day, the pre-culture was inoculated into the same medium at an initial OD_600_∼0.01 as the final experimental culture. The final experimental culture was first exponentially growing to OD_600_ ∼0.2; different doses of H_2_O_2_ were then added to induce oxidative stress. The OD_600_ of the culture was measured throughout the whole process to obtain a complete growth curve. Related parameters such as translational elongation rate, cellular tRNA abundance and total catalase activity were measured at specific time points. For cells harboring pFL-series vector, the cultures were always supplemented with 50-μg/ml ampicillin (Coolaber Biotech).

### Translational elongation rate measurement

Measurement of translational elongation rate was based on LacZ induction assay as well as GFP fusion protein induction assay (ManA-GFP and FusA-GFP). The LacZ induction assay with a 10-s initiation time calibration was performed the same as described in Dai *et al.* (25), as described in Supplementary Figure S3 and S4. The procedure of GFP induction assay was slight different from that of LacZ induction assay. In brief, 5 mM IPTG was added to the FL174 and FL175 culture to induce the expression of ManA-GFP or FusA-GFP. Immediately after addition of IPTG, at 10- to 30-s intervals (depends on the translational elongation rate), 20–30 aliquots of 300-μl cultures were transferred into pre-chilled microfuge tube containing 10-μl chloramphenicol (34 mg/ml) (Coolaber Biotech). The samples were kept on ice for over 6 h before measuring the GFP fluorescence. The GFP fluorescence intensity was measured by a micro-plate reader (485 nm excitation filter, 528 emission filters). The GFP induction curve was made by plotting the fluorescence intensity against the induction time and analyzed using the square root plot (Schleif plot). From the Schleif plot, we could obtain the time needed for the appearance of first round of GFP fusion protein, *T*_first_. The translational elongation rate was obtained using the GFP protein length to divide (*T*_first_ = 10 s), where the 10-s was the time cost of initiation steps. The length of ManA-GFP and FusA-GFP protein are 639 and 952 aa, respectively.

### Measurement of the GFP fluorescence units

A 300-μl GFP cell sample was directly pipetted into a pre-cooled 1.5 mL eppendorf tube containing 10-μl chloramphenicol (34 mg/ml) and further put on ice for 6 h before measuring fluorescence units. GFP fluorescence was measured with a synergy-2 micro-plate reader (Biotek) at 485-nm/528 nm Ex/Em mode. The GFP value of each sample was subtracted by the background fluorescence of wild type NCM3722 strain at the same OD_600_ point.

### Measurement of cellular individual tRNA and mRNA by qRT-PCR

The cellular tRNA abundance was measured by qRT-PCR. In brief, 0.8 ml of cell culture was transferred to a plastic tube containing 0.8 ml pre-cooled stop solution (60% ethanol, 2% phenol and 10 mM EDTA). The total cellular RNA was then extracted with a bacterial total RNA extraction kit (TianGen, China). The final concentration of RNA was then measured with a NanoDrop-1000 micro-spectrophotometer. 1-μg total cellular RNA sample was first pre-treated in 80°C for 15 min to remove the secondary structure of tRNA and immediately put into ice. The first-strand cDNA synthesis was performed using a first-strand cDNA synthesis reverse transcriptase kit (TianGen Biotech, China) using random primers. The qRT-PCR reaction was performed based on the Super-premix SYBR green Plus kit (Yeasen Biotech, Shanghai, China) using Bio-rad CFX96 Touch real-time PCR system. Detailed qRT-PCR reaction protocol was as follows: 95°C for 15 min, followed by 40 cycles of 95°C for 10 s, 60°C for 20 s, and 72°C for 30 s. The 5S rRNA was used as the internal references since rRNA concentration or total RNA concentration remains constant upon oxidative stress (35). The related qRT-PCR primers were based on ref. (35). The Ct value (for both tRNA and 5S rRNA) of the sample at each time point after H_2_O_2_ addition was obtained from the machine. The relative tRNA level of time 0 sample was set as ‘1’. Relative expression of tRNA at different time points was calculated according to the 2^−ΔΔCT^ method. The mRNA quantification of *oxyR*-regulon genes was similar with tRNA quantification except that the total cellular RNA did not require pre-treatment in 80°C before cDNA synthesis.

For measurement of the transcriptional kinetics of the full-length *lacZ* mRNA and *manA-gfp* mRNA shown in Supplementary Figure S6, *E. coli* cells were exponentially growing to OD_600_ ∼0.4 followed by the induction of *lac* operon or *Ptac-manA-gfp* expression through addition of 5 mM isopropyl-β-D-thiogalactoside (IPTG). Immediately after the IPTG induction, 0.8 ml of cell culture was taken at a 15-s interval and transferred into a pre-cooled plastic tube containing 0.8 mL stop solution containing 60% ethanol, 2% phenol and 10 mM EDTA. The RNA extraction and reverse transcription process was then performed the same as described above. Two pairs of qRT-PCR primers were used to detect the 3′-end region of *lacZ* mRNA and *manA-gfp* mRNA respectively. For *lacZ* mRNA, forward primer: GCACATGGCTGAATATCGACG; Reverse primer: P3105-R: GACACCAGACCAACTGGTAATGG. For *manA-gfp* mRNA, forward primer: TCCACACAATCTGCCCTTTCG; reverse primer: TGTGTAATCCCAGCAGCTGTTAC. The relative mRNA abundance in each time point equals to 2^Ct(0)-Ct(t)^, where Ct(0) means the Ct value of the sample taken immediately before IPTG addition and Ct(t) means the Ct value at each time point. The mRNA abundance was plotted with the time to obtain the transcriptional kinetics curve.

### Measurement of total catalase activity

The total catalase activity was performed similarly as described in (36). Catalase activity determination was based on measuring the rate of decomposition of hydrogen peroxide, which was proportional to the reduction of the absorbance at λ = 240 nm. Briefly, 10 mL *E. coli* cells were collected by centrifuge and suspended in 1.5 ml 50 mM phosphate buffer (pH 7.0). Cells were further lysed by sonication and centrifuged at 13 000 rpm at 4°C for 10 min, after which the supernatant was taken for catalase activity measurement. 0.1 ml crude extract was added to 0.9 ml phosphate buffer (pH 7.0); hydrogen peroxide was then added to a final concentration of 5 mM. The absorbance of the samples at 240 nm was measured every 30 s for 10 min by a micro-plate reader (Biotek). Catalase activities were further normalized by the total protein concentration of the crude extracts.

## RESULTS

### Growth arrest of *E. coli* cells upon H_2_O_2_ shock

**Figure 1.**
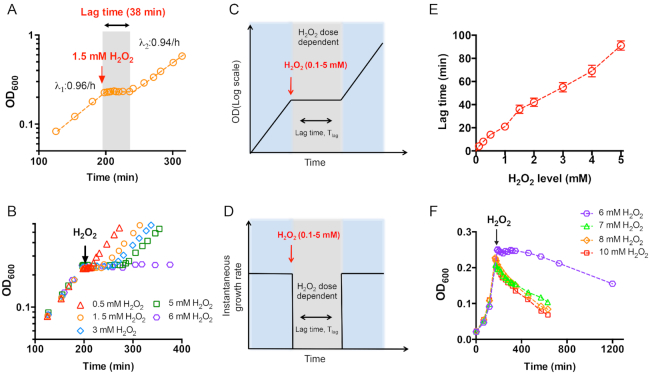
Growth of *E. coli* cells subjecting to the treatment of different doses of hydrogen peroxide (H_2_O_2_). (**A**) Growth curve of *E. coli* cells in glucose minimal medium treated with 1.5 mM H_2_O_2_. Cells were first exponentially growing to OD_600_∼0.2; 1.5 mM H_2_O_2_ was then added (red arrow) and cell growth immediately stopped. After a lag time of 38 min (gray part), cell growth recovered to normal speed. (**B**) Growth curve of *E*. coli cells in glucose minimal medium treated with various levels of H_2_O_2_ (from 0.5 to 6 mM). The full range of 6 mM data is shown in panel F. (**C, D**) Growth pattern of *E. coli* cells upon treatment of 0.1 to 5 mM H_2_O_2_. Cells were first exponentially growing at a constant growth rate (panel D), H_2_O_2_ (red arrow) was then added and cell growth was immediately arrested (panel C). At this stage, instantaneous growth rate dropped into zero (panel D). After a certain lag time, cell growth completely recovered (panel C). The growth rate after growth recovery was the same as that before H_2_O_2_ treatment. The lag time is positively dependent on H_2_O_2_ dose. (**E**) The correlation between the lag time and the H_2_O_2_ dose. (**F**) Growth curve of *E. coli* cells in glucose minimal medium treated with ≥6 mM H_2_O_2_.

We focus on the effect of H_2_O_2_ treatment on the wild type *E. coli* K-12 strain (NCM3722) exponentially growing in glucose medium (Figure 1A). *Escherichia coli* cells were first growing exponentially in glucose minimal medium at a growth rate of λ_1_: 0.96/h. When the optical density of the culture at 600 nm (OD_600_) reached ∼0.2, 1.5 mM H_2_O_2_ was added to impose oxidative stress on the cell culture (red arrow of Figure 1A); cell growth was immediately arrested into zero. After a lag time of 38 min (gray region of Figure 1A), cell growth completely recovered to its normal state, λ_2_: 0.94/h, indicating that the excess intracellular H_2_O_2_ had been successfully removed during the lag time. This phenomenon of transient growth arrest of *E. coli* cells upon H_2_O_2_ shock is consistent with previous literatures focusing on *E. coli* cells, yeast cells and animal cells (14). Earlier studies had found that *E. coli* K-12 cells could smoothly survive and resume normal growth upon 2 mM H_2_O_2_ shock, which is consistent with our finding (37,38).

We next repeated the same growth experiments at different doses of H_2_O_2_ (the different symbols in Figure 1B and Supplementary Figure S1). At the range of 0.1–5 mM H_2_O_2_, the general growth patterns of *E. coli* cells were similar at different H_2_O_2_ levels. Growth was immediately arrested but completely recovered after a certain lag time (Figure 1B–D). However, the lag time depends positively on the external H_2_O_2_ levels (Figure 1E). The lag time was only 5–15 minutes at low H_2_O_2_ range (0.1–0.5 mM, Figure 1E and Supplementary Figure S1), but dramatically increased to ∼90 min at 5 mM H_2_O_2_ (green square in Figure 1B and E). During the lag time, the cell viability was not affected at all (Supplementary Figure S2), indicating that cells could tolerate potential oxidative damage for a period of time. At 6 mM H_2_O_2_, cell growth was also immediately arrested (purple hexagon in Figure 1B and F). However, cell mass kept constant for ∼3 h and then dropped gradually (purple hexagon in Figure 1F), indicating that 6 mM is the threshold H_2_O_2_ level at which *E. coli* cells ultimately fail to tolerate. When the external H_2_O_2_ levels were further increased, the cell mass could not be sustained and decreased quickly (Figure 1F). In summary, as schematically illustrated in Figure 1C and D, cell growth is immediately arrested at a certain range of H_2_O_2_ levels (0.1–5 mM). After a certain lag time (depending positively on the H_2_O_2_ dose), cell growth completely recovers. However, when the external H_2_O_2_ level is higher than the threshold level (6 mM), cells ultimately fail to survive and resume growth.

### Time-course analysis of translational elongation rate and cellular tRNA pools upon H_2_O_2_ shock

There are two important observations in the above section: (i) At the range of 0.1-5 mM H_2_O_2_ level, the lag time depends positively on the H_2_O_2_ levels, and (ii) *E. coli* fails to tolerate a certain high level of H_2_O_2_ (≥6 mM). These findings pose a fundamental question: what's the physiological bottleneck that limits the survival of bacterial cells during oxidative stress? If it is simply an issue that higher levels of H_2_O_2_ require more time to be removed by catalases, it is difficult to understand why *E. coli* cells could completely resume normal growth at 5 mM H_2_O_2_ but fail to tolerate a slightly higher H_2_O_2_ level of 6 mM.

**Figure 2.**
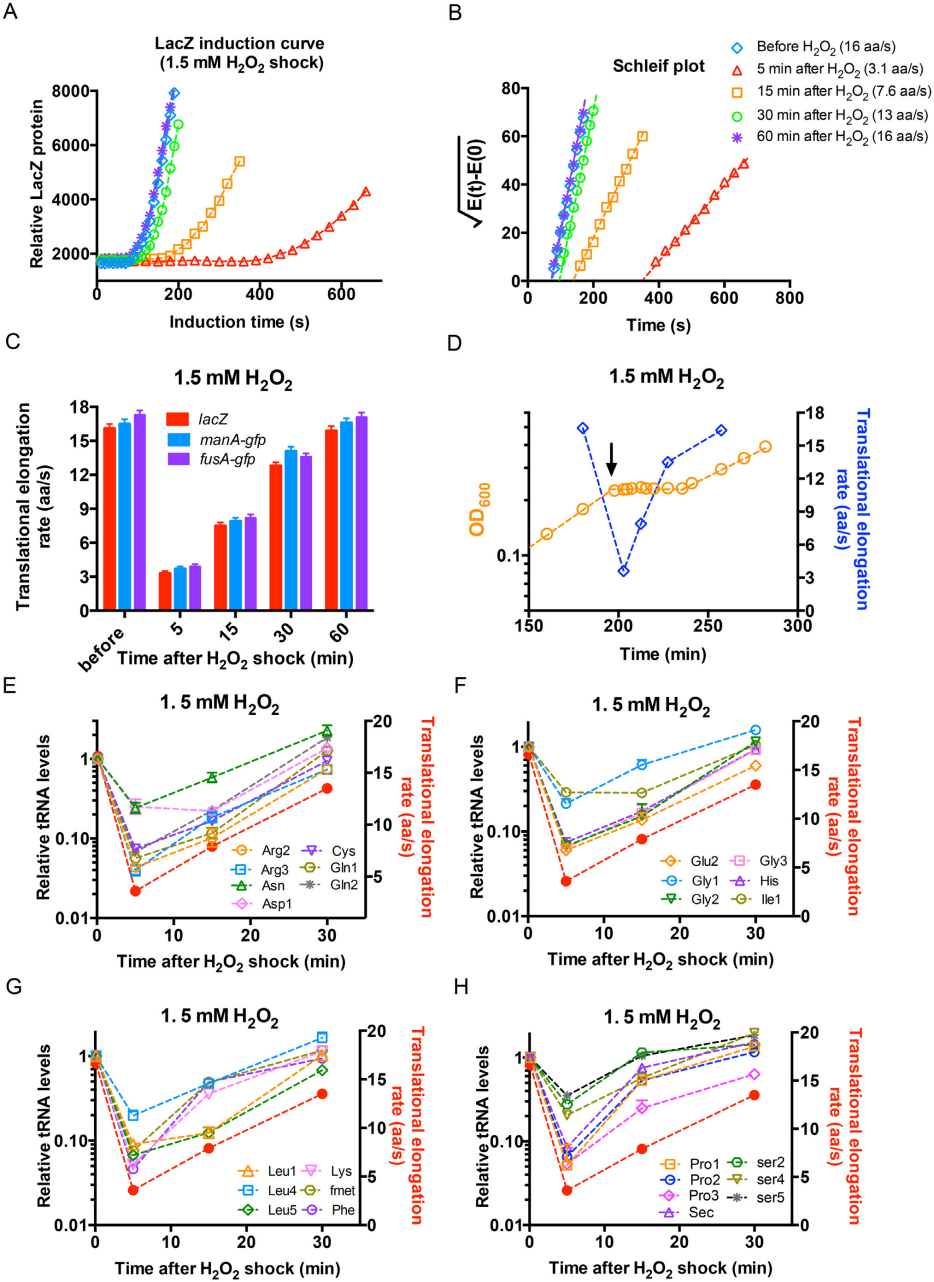
The translational elongation rate and cellular tRNA pools of *E. coli* subjecting to 1.5 mM H_2_O_2_ treatment. (**A**) The induction curves of LacZ protein for *E. coli* NCM3722 strain growing in glucose minimal medium at various time points (5, 15, 30, 60 min) after the addition of 1.5 mM H_2_O_2_. The same experiment was also conducted for culture before the addition of H_2_O_2_. (**B**) The Schleif plot of the LacZ induction curve shown in panel A. (**C**) The translational elongation rate of three genes of *E. coli* cells before H_2_O_2_ treatment as well as at various time points (5 min, 15 min, 30 min, 60 min) after the addition of 1.5 mM H_2_O_2_. (**D**) The translational elongation rate is shown together with the growth curve of *E. coli* cells subjecting to 1.5 mM H_2_O_2_ treatment. The data of translational elongation rate is the average of the values of three genes shown in panel C. (**E**–**H**) The cellular tRNA pools of *E. coli* cells at various time points (0, 5, 15, 30 min) after the addition of 1.5 mM H_2_O_2_. The translational elongation rates at those time points were shown as red solid symbols. The data at time 0 for each individual tRNA species is set as 1. The standard deviations of triplicates are shown but are very small in the log-scale plot.

H_2_O_2_ shock activates the *oxyR* regulon of *E. coli* cells, inducing the expression of related genes such as *ahpCF, katG and trxC*, which enable bacterial cells to scavenge the intracellular H_2_O_2_, maintain redox homeostasis and resume cell growth. The *de novo* synthesis of stress defense proteins can be strongly affected by the overall protein translational elongation status of *E. coli*. Recent indirect evidence such as polysome profiling, has indicated that oxidative stress could inhibit the translational elongation of *E. coli* (35). However, it remains unclear to what extent the translational elongation is suppressed *in vivo*, and, moreover, the effect of translation elongation status on the oxidative defense response. We next quantitatively characterized the ribosome translational elongation rate (ER) of *E. coli* during H_2_O_2_ treatment. The ER was measured using the classical β-galactosidase (LacZ) induction assay (Supplementary Figure S3) (25) with correction of a 10-s time cost for initiation steps (Supplementary Figure S4). The LacZ induction assay was performed at various time points before and after the addition of 1.5 mM H_2_O_2_ (Figure 2A). Under the normal growth condition, the ER value was high, ∼16 aa/s (blue diamond in Figure 2B). Strikingly, at 5 min after the addition of 1.5 mM H_2_O_2_, the synthesis of full-length LacZ protein took a much longer time (5-fold) than it did under the normal condition, ∼350 s (red triangle of Figure 2B), corresponding to an ER of only ∼3 aa/s (Figure 2C, red bar). This result directly supports that the oxidative stress strongly inhibits the translational elongation process. ER then gradually recovered and reached its normal state at 60 min after the onset of H_2_O_2_ treatment (Figure 2C). To rule out the possibility that our result is specific to LacZ translation, we also measured the ER of two GFP fusion proteins, ManA-GFP and FusA-GFP, upon H_2_O_2_ treatment (Supplementary Figure S5). The time-course behavior of ER of these two proteins is quantitatively consistent with that of LacZ protein (Figure 2C). By plotting the growth curve and ER in the same plot (Figure 2D), it can be clearly seen that ER dramatically decreased at the initial stage (∼5 min) of H_2_O_2_ shock, but gradually recovered during the lag time and reached a normal value shortly after growth recovery. To investigate whether the slow-down of translational elongation was related to a slow-down of mRNA transcription, we measured the induction kinetics of *lacZ* mRNA and *manA-gfp* mRNA of *E. coli* cells at 5 min after addition of 1.5 mM H_2_O_2_ using qRT-PCR. As shown in Supplementary Figure S6 (blue circles), the transcription of full-length *lacZ* mRNA and *manA-gfp* mRNA was strongly induced by IPTG addition and required a much shorter time (∼170 s for *lacZ* mRNA) to synthesize than the full-length proteins. Therefore, the translational ER under oxidative stress was not limited by the mRNA transcriptional elongation.

A fast ER requires highly abundant intracellular tRNA pools (25,39). It is known that substantial degradation of full-length tRNA occurs during oxidative stress and other stress conditions for many types of cells (35,40–43). Therefore, the dramatic slow-down of translational elongation upon H_2_O_2_ shock might be due to lower cellular tRNA pools. We next used qRT-PCR to quantitatively characterize the time-course behavior of 26 individual tRNA levels of *E. coli* cells during H_2_O_2_ shock. Substantial alteration in cellular tRNA pools was observed shortly after the onset of H_2_O_2_ shock. At 5 min, the abundance of most investigated tRNA species was reduced by ∼90% (Figure 2E–H). Moreover, similar to the trend of ER (solid red circles in Figure 2E–H), tRNA pools also gradually recovered later during the lag time (Figure 2E–H). In summary, the alterations in tRNA pools correlate well with the dramatic change in ER during H_2_O_2_ shock.

### H_2_O_2_-dose dependent translational elongation rate and cellular tRNA pools

**Figure 3.**
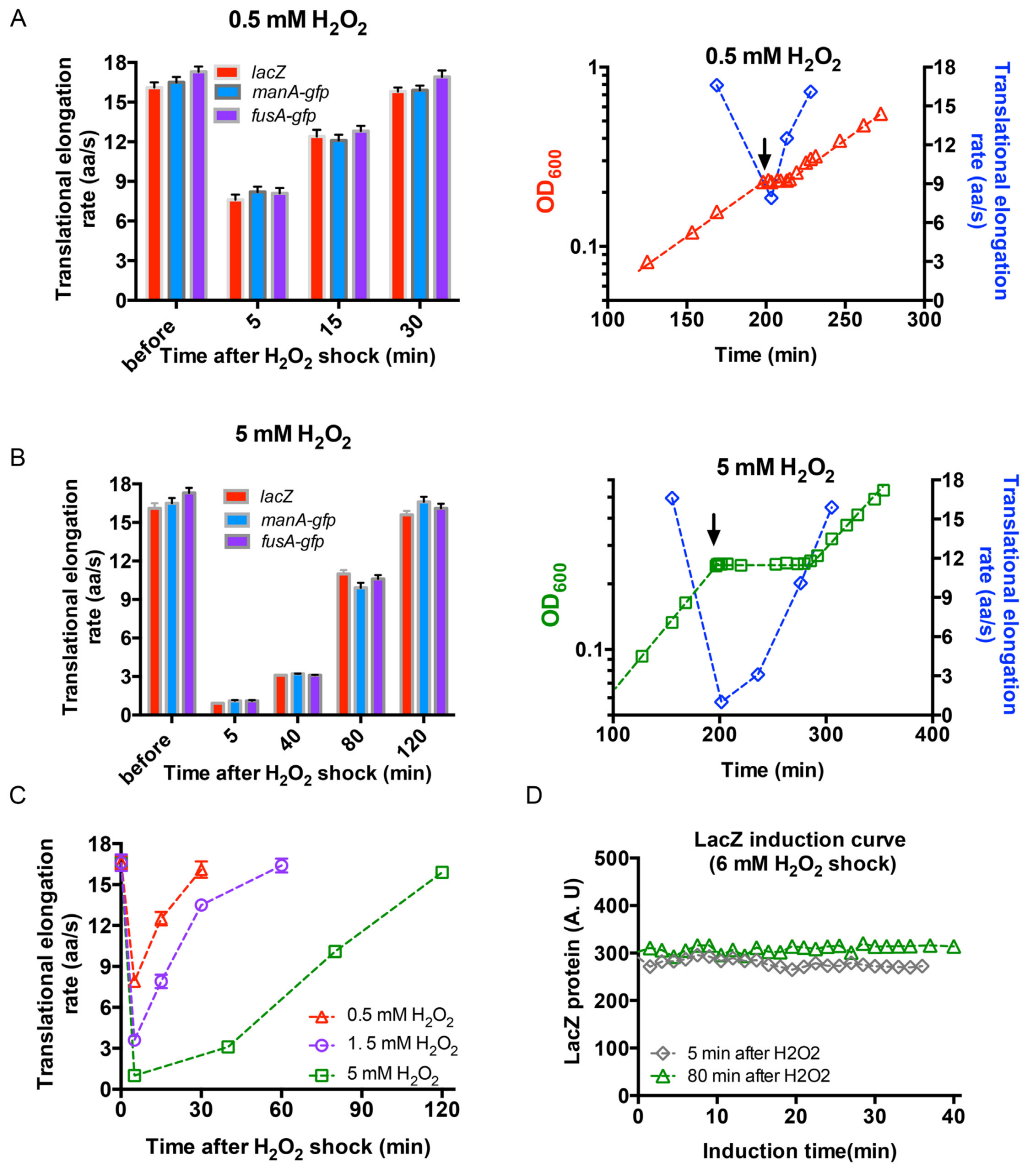
The translational elongation rate of *E. coli* subjecting to 0.5, 5 and 6 mM H_2_O_2_ treatment. (**A**) The translational elongation rate of three genes of *E. coli* cells before treatment as well as at various time points (5, 15, 30 min) after the addition of 0.5 mM H_2_O_2_. At the right panel, the translational elongation rate is shown together with the growth curve of *E. coli* cells subjecting to 0.5 mM H_2_O_2_ treatment. The data of translational elongation rate is the average of the values of three genes. (**B**) The translational elongation rate of three genes of *E. coli* cells before H_2_O_2_ treatment as well as at various time points (5, 40, 80 and 120 min) after the addition of 5 mM H_2_O_2_. At the right panel, the translational elongation rate is shown together with the growth curve of *E. coli* cells subjecting to 5 mM H_2_O_2_ treatment. The data of translational elongation rate is the average of the values of three genes. (**C**) The translational elongation rate of *E. coli* at various times points after the addition of 0.5, 1.5 and 5 mM H_2_O_2_. The data at time 0 corresponds to the normal value before H_2_O_2_ addition. (**D**) The induction curve of LacZ protein for *E. coli* cells growing in glucose minimal medium at two time points (5 min and 80 min) after the addition of 6 mM H_2_O_2_.

To gain a more systematic insight into the effect of H_2_O_2_ shock on ER and cellular tRNA pools, we repeated the above study with a lower H_2_O_2_ level (0.5 mM) and a higher H_2_O_2_ level (5 mM) (Figure 1B and E). In the case of a 0.5 mM H_2_O_2_ shock, we measured the ER at 5, 15 and 30 min after the addition of H_2_O_2_ (Figure 3A, left panel). At 5 min after a 0.5 mM H_2_O_2_ shock, ER dropped by 50%, to ∼8 aa/s, which was significantly higher than the value in the case of a 1.5 mM H_2_O_2_ shock (∼3 aa/s). Furthermore, ER recovered to its normal value at 30 min (Figure 3A, right panel), which was shorter than the time required for ER recovery in the case of 1.5 mM H_2_O_2_ treatment. We also measured the ER at 5, 40, 80 and 120 min after the addition of 5 mM H_2_O_2_. At 5 min, the synthesis of full-length LacZ protein took nearly 20 min (Supplementary Figure S7A), corresponding to an extremely slow ER of 1 aa/s, which was also found for ManA-GFP and FusA-GFP protein (Figure 3B, left panel; Supplementary Figure S7B and C). Although ER also gradually recovered latter, the recovery rate was much slower than that in the case of 1.5 and 0.5 mM H_2_O_2_ treatment (Figure 3C). As shown in Figure 3B, the ER increased to only ∼3 aa/s at 40 min and remained sub-optimal, at 10 aa/s, at the end of lag time (80 min) (Figure 3B, left panel). Those results show that the stress on translational elongation dramatically aggravates with increasing H_2_O_2_ levels. From this perspective, we expected that translational elongation might stall completely at an even higher H_2_O_2_ dose. We thus performed the LacZ induction assay at 6 mM H_2_O_2_, in which cells fail to survive and resume growth (Figure 1F). At both 5 and 80 min after the onset of 6 mM H_2_O_2_ shock, no synthesis of new LacZ protein was observed after an induction time of 40 min (Figure 3D). The same result was observed in the case of ManA-GFP and FusA-GFP proteins (Supplementary Figure S8), supporting that translational elongation process is completely arrested at 6 mM H_2_O_2_ (ER: 0 aa/s).

**Figure 4.**
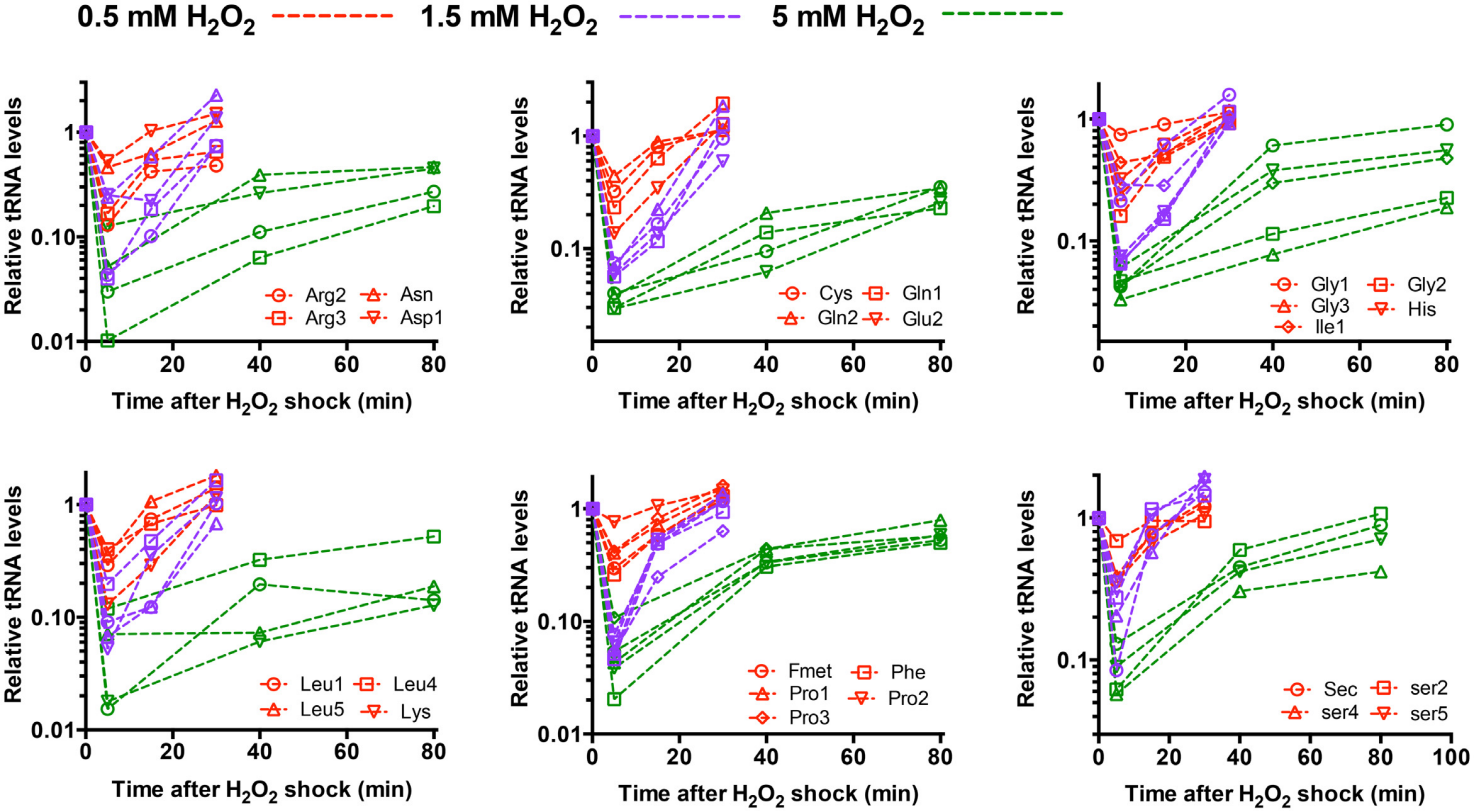
The relative cellular tRNA abundances of *E. coli* at various times points after the addition of 0.5, 1.5 and 5 mM H_2_O_2_. The data at time 0 for each individual tRNA species is set as 1. The standard deviations of triplicates are shown but are very small in the log-scale plot.

The time-course behavior of cellular tRNA pools of *E. coli* cells (Figure 4) exhibited a similar time-course pattern as ER (Figure 3C). However, the extent of the alteration of tRNA pools exhibited a strong H_2_O_2_-dose dependence. The drop in cellular tRNA pools at 5 min after addition of 0.5 mM H_2_O_2_ was remarkable but much milder than the drop after addition of 1.5 mM H_2_O_2_. In contrast, the abundances of most tRNA species dropped by 95% at 5 min after addition of 5 mM H_2_O_2_ (green symbol in Figure 4), which was a more severe drop than that of addition of 1.5 mM H_2_O_2_. Therefore, the severity of the stress on translational elongation at different H_2_O_2_ levels was largely determined by the cellular tRNA pools.

### Severely delayed synthesis of oxidative stress-defense proteins at high H_2_O_2_

**Figure 5.**
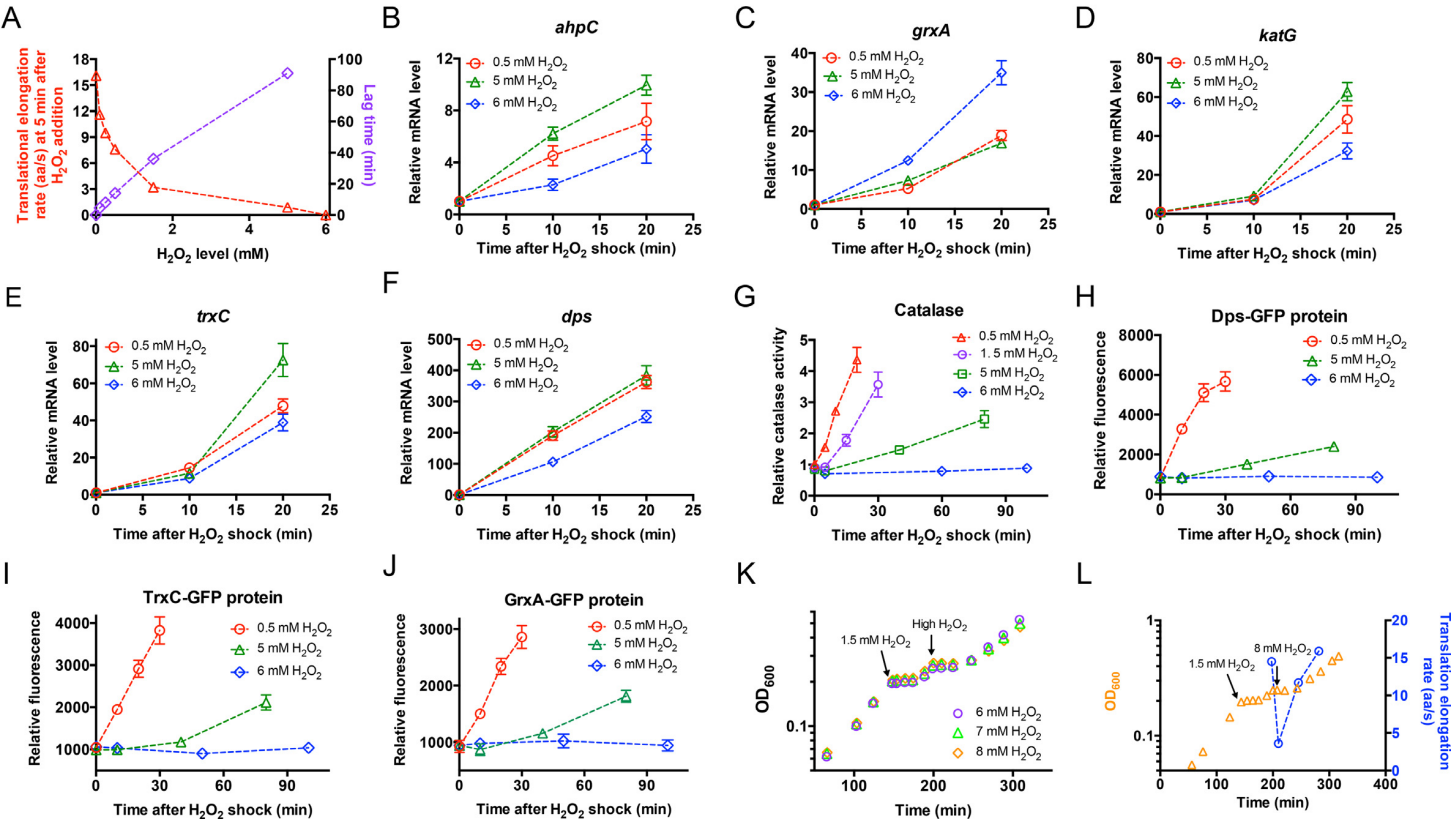
Inhibition of translational elongation by oxidative stress limits the de novo synthesis of catalase. (**A**) The correlation of translational elongation rate (at 5 min after the addition with H_2_O_2_) and lag time with the dose of H_2_O_2_. (**B–F**) The mRNA levels of five genes in *oxyR*-regulon at various time points after the addition of 0.5, 5 and 6 mM H_2_O_2_. (**G**) The total catalase activity of *E. coli* at various time points after the addition of 0.5, 1.5, 5 and 6 mM H_2_O_2_. The data at time zero of 0.5 mM H_2_O_2_ is set as 1. (**H–J**) The levels of three GFP-fused proteins at various time points after the addition of 0.5 mM, 5 mM and 6 mM H_2_O_2_. (**K**) Pre-adaption of a low H_2_O_2_ dose allows cell to counteract a higher lethal H_2_O_2_ dose than no pre-adapted cells. *E. coli* cells were first subject to the treatment of 1.5 mM H_2_O_2_; after the growth recovery, high doses of H_2_O_2_ (6, 7 and 8 mM) were respectively added to the cultures to initiate a second round of oxidative stress. (**L**) The translational elongation rate and growth curve of *E. coli* cells (pre-adapted to 1.5 mM H_2_O_2_ treatment) upon subjecting to 8 mM H_2_O_2_ treatment.

The above result demonstrates that *E. coli* cells face severe problem in maintaining the ER during oxidative stress, which may significantly compromise the process of cellular stress response to oxidative stress. When confronting H_2_O_2_ shock, bacterial cells need to immediately initiate stress defense programs such as the *oxyR* regulon to defend them against H_2_O_2_ stress. The *oxyR* regulon lies at the core of bacterial defense against oxidative stress. The *oxyR*-null strain, which fails to induce the expression of related oxidative defense proteins, becomes hypersensitive to H_2_O_2_ treatment (20,22,23,44). It is known that oxidative defense genes such as *ahpCF, katG* are maximally induced within as short as ∼10 min at a low dose of H_2_O_2_ (18,22). However, the stress on translational elongation at high H_2_O_2_ levels might significantly delay the timely synthesis of those oxidative defense proteins. For example, ER is only ∼1 aa/s at 5 min after the addition of 5 mM H_2_O_2_, in which case it would take ∼12 min to translate a full-length KatG protein (726 amino acids). As shown in Figure 5A, the increase in lag time required for growth recovery coincided strongly with the decrease of ER at 5 min after H_2_O_2_ addition. Specially, 6 mM was the threshold H_2_O_2_ level at which ER drops to zero and cells fail to resume growth.

We next directly investigated whether the stress on translational elongation upon H_2_O_2_ treatment could also inhibit the synthesis of oxidative defense proteins. We first measured the mRNA levels of five genes in *oxyR*-regulon, *ahpC, grxA, katG, trxC* and *dps*, at three H_2_O_2_ levels. As shown in Figure 5B-F, the mRNA levels of all five genes strongly increased regardless of the level of H_2_O_2_, indicating that the *oxyR* regulon was strongly activated in the transcriptional level upon H_2_O_2_ shock. We then investigated the synthesis of catalase upon H_2_O_2_ treatment. The *E. coli* cells have two major catalases, KatG (HPI) and KatE (HPII). Although *katE* is not induced by H_2_O_2_ shock, *katG* is strongly induced by OxyR upon H_2_O_2_ shock (17,18). Therefore, low doses of H_2_O_2_ should remarkably elevate the total catalase activities of *E. coli* cells. As shown in Figure 5G, the total catalase activity of *E. coli* indeed strongly increased by several-fold within 30 min at two low levels of H_2_O_2_ (red triangle and purple circles). However, the process of catalase accumulation became much slower in *E. coli* cells treated by 5 mM H_2_O_2_ (green squares at Figure 5G). When the H_2_O_2_ level reached 6 mM, no catalase accumulation was observed within 100 min (blue diamond, Figure 5G), which would be as expected if translational elongation of oxidative defense proteins also gets completely stalled as observed in the cases of LacZ and GFP proteins. To see whether other oxidative defense proteins exhibit similar behaviors as catalases, we focused on three additional OxyR-regulated proteins, Dps, TrxC and GrxA. We directly fused the three proteins with GFP protein and placed the three translational-fusion GFP proteins downstream of their native OxyR-regulated promoters. The synthesis of all the three proteins were also strongly induced at low H_2_O_2_ level (red circles in Figure 5H–J) but severely inhibited or completely abolished at 5 or 6 mM H_2_O_2_, respectively. Overall, the stress on translational elongation upon H_2_O_2_ shock could indeed limit the timely synthesis of oxidative defense proteins.

At low levels of H_2_O_2_, *E. coli* cells could smoothly induce the *oxyR*-defense program and accumulate related defense proteins. If it is indeed the inhibition of the timely synthesis of oxidative defense proteins that underlies the failure of *E. coli* in tolerating high levels of H_2_O_2_, we expect cells pre-adapted in a low dose of H_2_O_2_ could acquire increased H_2_O_2_ tolerance. To test this scenario, we first had the exponentially growing *E. coli* treated with 1.5 mM H_2_O_2_; after the recovery of growth, we added 6 mM, 7 mM and 8 mM H_2_O_2_ to the cell culture (Figure 5K). Strikingly, *E. coli* cells could resume growth in all the three high H_2_O_2_ levels after a ∼30 min lag time (Figure 5K). Moreover, ER of pre-adapted cells was still significant (3.6 aa/s) at 5 min after 8 mM H_2_O_2_ shock and again recovered into the normal value later (blue circles in Figure 5L). Therefore, if cells have pre-accumulated stress-related proteins, translation stress would be substantially alleviated and the cells could overcome a higher H_2_O_2_ dose than cells without pre-adaptation.

### tRNA-limited translational elongation strongly affects the growth fitness during oxidative stress

**Figure 6.**
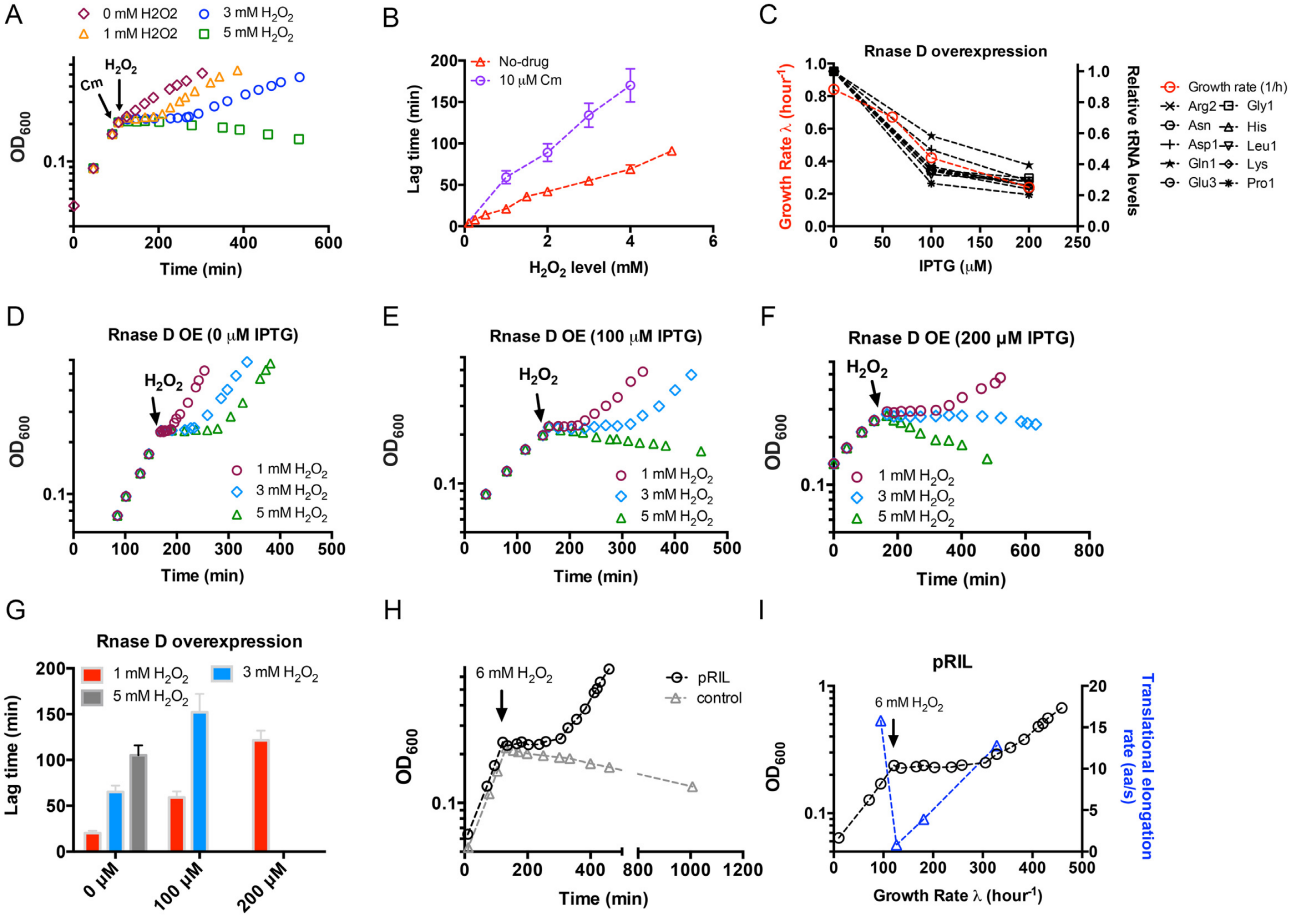
The tRNA-limited translational elongation process significantly affects cell fitness during oxidative stress. (**A**) Growth curve of chloramphenicol (Cm)-treated *E. coli* cells being subjected to H_2_O_2_ treatment. Cells were first exponentially growing in glucose medium to OD600 ∼0.2, 10 μM Cm was then added to inhibit the translation process. After 5 min, different doses of H_2_O_2_ were added to the cultures. (**B**) The correlation between the lag time and the H_2_O_2_ doses for cells treated with or without Cm. (**C**) The growth rate and individual tRNA levels of *E. coli* upon different degrees of Rnase D overexpression. The expression of Rnase D was controlled by the IPTG-inducible *Ptac* promoter. (**D–F**) Growth curve of H_2_O_2_-treated *E. coli* cells upon different degrees of Rnase D overexpression. (**G**) Lag time of H_2_O_2_-treated *E. coli* cells upon different degrees of Rnase D overexpression. (**H**) Effect of tRNA upregulation by pRIL plasmid on the growth of *E. coli* cells subjecting to 6 mM H_2_O_2_ treatment. The control group corresponds to *E. coli* cells harboring pACYC184 plasmid (with the same chloramphenicol resistance marker and p15A origin as pRIL). (**I**) Translational elongation rate of *E. coli* cells harboring pRIL plasmid during 6 mM H_2_O_2_ treatment. The growth curve of panel D is shown here.

The stress on translational elongation, which limits the timely synthesis of stress defense proteins, is likely to impose a severe physiological burden on *E. coli* during counteracting oxidative stress. To further test the above notion, we performed three experiments. In the first experiment, we added a sublethal dose (10 μM) of chloramphenicol (Cm) to the exponentially growing culture to inhibit protein translation. Cm is a bacteriostatic antibiotic that blocks the elongation process of bacterial translation without affecting cell viability (45,46). At 5 min after the addition of Cm, we treated the cells with different doses of H_2_O_2_ (Figure 6A). The growth rate of *E. coli* cells at 10 μM Cm (doubling time: 170 min) was only one fourth of that at no-drug condition (doubling time 43 min) so that the overall translational rate dropped by 75% (Figure 6A, magenta diamonds). We found that the Cm-treated cells could not tolerate 5 mM H_2_O_2_ (Figure 6A, green squares). Moreover, the lag times of Cm-treated cells at lower H_2_O_2_ doses (1–4 mM) were much longer than that of drug-free cells (Figure 6B). These results indicate that decreased translational rate indeed affect cell fitness upon oxidative stress.

In the second experiment, we attempted to artificially enlarge the tRNA translational stress of *E. coli* cells and test their fitness during oxidative stress. The Rnase D protein (47,48), which has high specificity to tRNA, was placed downstream of the IPTG-inducible *Ptac* promoter. It is known that overexpression of Rnase D proteins could effectively down-regulate cellular tRNA pools and reduce cell growth rate (48–50), as confirmed by our measurements of individual tRNA pools and growth rate (Figure 6C). We next characterized cell growth upon H_2_O_2_ treatment upon different degrees of Rnase D overexpression. At 0 μM IPTG level, cells could smoothly survive and resume growth at all three H_2_O_2_ levels (Figure 6D), which was similar with the behavior of wild type cells. However, at 100 μM IPTG level, cells could not tolerate 5 mM H_2_O_2_ (green triangles in Figure 6E), and had a much longer lag time at 1 and 3 mM H_2_O_2_ than they did at no IPTG condition (red and blue bars in Figure 6G). The situation became even more severe for the condition of 200 μM IPTG, at which cells failed to tolerate 3 mM H_2_O_2_ (Figure 6F). In addition, the lag time of cells treated by 1 mM H_2_O_2_ at 200 μM IPTG level increased to as long as ∼2 h, which was 6-fold of no-IPTG condition (red bars in Figure 6G). In contrast, cells overexpressing a control protein, ManA-GFP, could still smoothly resume growth at all three H_2_O_2_ levels (Supplementary Figure S9). Those observations, being qualitatively consistent with the result of Cm treatment, reinforce the notion that translation stress severely limit cell survival during oxidative stress.

Finally, we transformed a tRNA up-regulated plasmid, pRIL (51), into *E. coli* cells. pRIL plasmid harbors extra copies of related rare tRNA species including *argU* encoding tRNA4^Arg^ (AGA, AGG), *ileY* encoding tRNA2^Ile^ (AUA) and *leuW* encodes tRNA3^Leu^ (CUA) (51). The *E. coli* cells harboring pRIL could indeed overcome 6 mM H_2_O_2_ after a certain lag time (∼ 2h) as shown in Figure 6H (black circle). In contrast, *E. coli* cells harboring the control plasmid still could not survive over 6 mM H_2_O_2_ treatment as found for wild type cells (Figure 1F). Throughout the whole process of H_2_O_2_ shock, translational elongation rate of pRIL-harboring cells was 0.8 aa/s at 5-min time point and again recovered to normal status during the recovery of cell growth (Figure 6I), being similar with the pattern of wild type cells during sublethal H_2_O_2_ treatment (0.5 to 5 mM). Overall, those various pieces of evidences support the scenario that tRNA-limited translational elongation process, being crucial for timely synthesis of stress defense proteins, becomes a key physiological bottleneck that limits the survival of *E. coli* cells during oxidative stress.

## DISCUSSION

Timely synthesis of stress defense proteins is important for *E. coli* to counteract stress conditions. In this work, we quantitatively characterize the growth of *E. coli* cell during H_2_O_2_-induced oxidative stress. We show that cells undergo temporary growth arrest at 0.1-5 mM H_2_O_2_ but completely resume normal growth after a certain lag time. The lag time is positively correlated with the dose of external H_2_O_2_. However, when the H_2_O_2_ dose reaches a threshold concentration of 6 mM, cells ultimately fail to survive and resume growth. This phenomenon poses an important question regarding the physiological bottleneck that limits bacterial fitness during oxidative stress. It has been known before that extensive tRNA degradation by Rnase occurs under oxidative stress in both bacterial cells and eukaryotic cells (42), however, its physiological consequence is not well understood. Here, we systematically combine the quantitative characterizations of tRNA and translational elongation rate of *E. coli* and establish their time-course behavior under various H_2_O_2_ levels. Surprisingly, we found that the decrease in tRNA pools is most substantial during the initial stage (∼5 min) of oxidative stress. At 5 min after the onset of 5 mM H_2_O_2_ shock, the abundances of most cellular tRNA species drop by ∼95%, leading to an unusually dramatic slow-down of translational elongation process (∼1 aa/s). The extent of the drop in ER during oxidative stress (1 aa/s at ∼ 5 min after the onset of 5 mM H_2_O_2_ shock) is much more remarkable than that found during any other kinds of stress conditions (e.g. nutrient deprivation, osmotic stress) (25–27). The severity of translational elongation stress positively depends on the level of H_2_O_2_. At the threshold H_2_O_2_ level of 6 mM, translation is even completely stalled.

**Figure 7.**
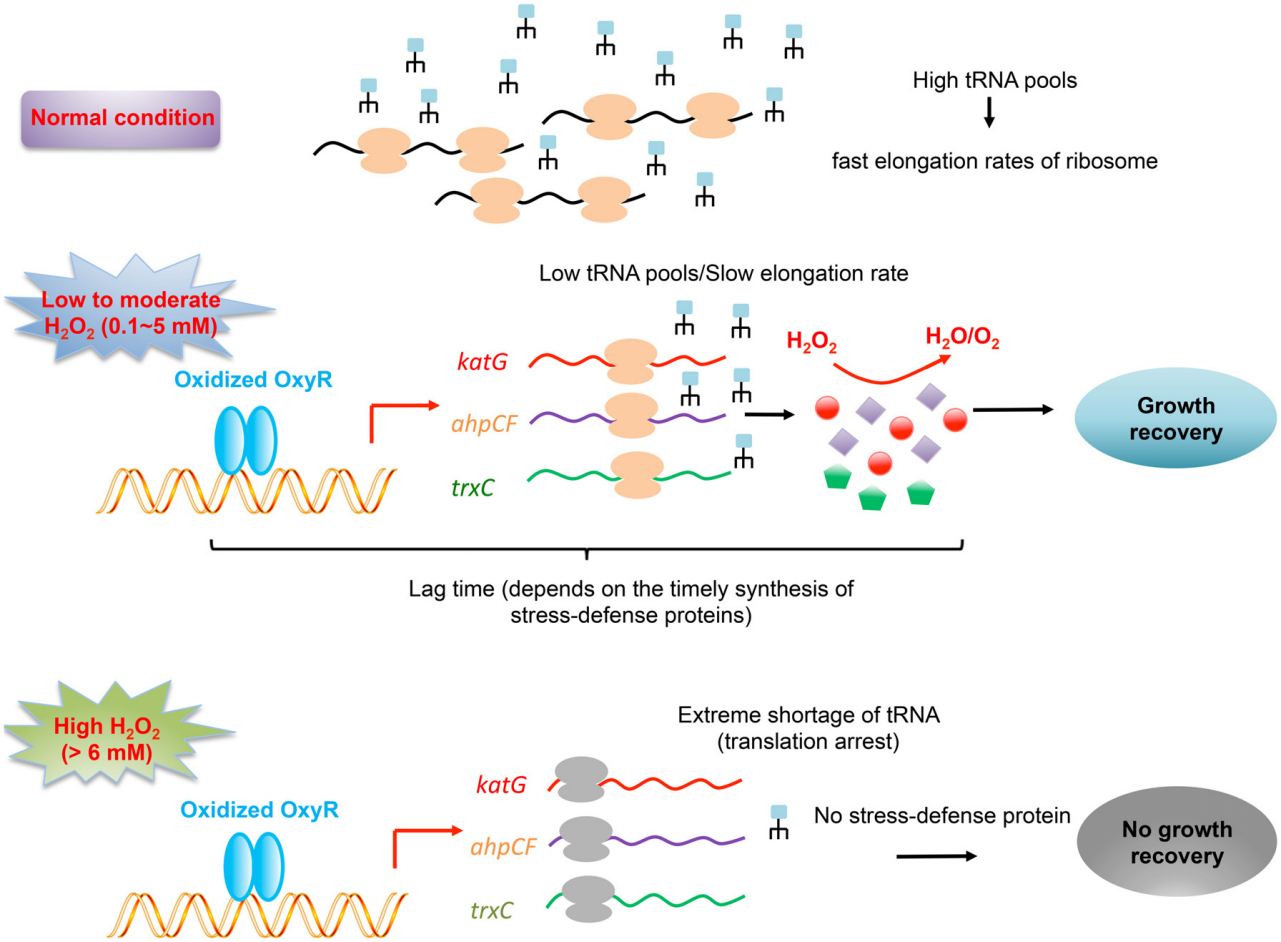
Schematic illustration depicting the maintenance of translational elongation rate as the physiological bottleneck limiting the survival of *E. coli* cells during oxidative stress. At normal condition, the cellular tRNA pools are highly abundant, supporting fast translational elongation rate of ribosomes. Upon the treatment of 0.1–5 mM H_2_O_2_, translational elongation significantly slows down due to lower tRNA pools. Therefore, cells need a certain lag time (depends on the H_2_O_2_ dosage) to synthesize stress defense proteins (e.g. KatG, AhpCF and TrxC) to remove H_2_O_2_ and maintain redox homeostasis before the growth can be resumed. When the external H_2_O_2_ level is higher than a threshold level (6 mM), translational elongation gets completely stalled so that cells lose the capacity to synthesize stress-defense proteins and ultimately fail to survive from the oxidative damage and resume growth.

Those findings support a scenario in which the tRNA-limited translational elongation process is a key physiological bottleneck that the cells need to overcome in order to smoothly survive and counteract H_2_O_2_ shock. As depicted in Figure 7, at normal growth condition, the highly abundant cellular tRNA pools support a high translational elongation rate; at 0.1–5 mM H_2_O_2_, the ribosome translational elongation slows down due to lower cellular tRNA pools; a higher H_2_O_2_ dose causes slower translational elongation rate so that cell needs a longer time to synthesize stress-defense proteins such as KatG, AhpCF and TrxC to get rid of H_2_O_2_ and maintain cellular redox homeostasis. Therefore, cells treated by a high level of H_2_O_2_ have a longer lag time for growth recovery than cells treated with a low level of H_2_O_2_. However, when external H_2_O_2_ level is higher than 6 mM, cells fail to synthesize related stress defense proteins due to complete arrest of translational elongation and thus cannot tolerate the oxidative damage (e.g. damage on nucleic acids, lipids and protein) from excess H_2_O_2_. The time-course behavior of oxidative stress-defensive proteins under different H_2_O_2_ provides direct evidence for the above picture (Figure 5G–J). In addition, artificial aggravating the situation of translation stress by either Cm treatment or Rnase D overexpression could further compromise the fitness of cells upon oxidative stress (Figure 6A–G). On the contrary, artificial overexpression of tRNA could alleviate the translational elongation stress and enable cells to better tolerate H_2_O_2_ treatment (Figure 6H and I).

The H_2_O_2_-mediated oxidative stress presents a striking example in which bacterial cells face severe problem in maintaining the elongation rate of ribosomes and further timely synthesis of stress defense proteins. Although stress defense programs such as *oxyR*-regulon have been strongly activated in the transcription level by H_2_O_2_ shock, it was significantly inhibited or completely abolished by high H_2_O_2_ level due to the severely compromised translational capacity. Upon stress conditions, timely synthesis of stress defense proteins depends on two crucial parameters, number of actively translating ribosomes (determined by translational initiation) and ER (the speed of ribosomes) (25,26). Since stress defense proteins are preferentially expressed under stress conditions, the number of actively ribosomes translating stress defense proteins is guaranteed. However, for ER, our study shows that the case of oxidative stress is quite different from that of nutrient starvation conditions (carbon starvation and amino acid starvation) since at the later case, *E. coli* still maintains a significant ER (8–9 aa/s) so that timely synthesis of related stress defense protein is guaranteed (25,27,30). Although cellular tRNA levels also drop significantly during amino acid starvation due to degradation (43,52), the drop seems to be much milder than the case of H_2_O_2_ shock. This may account for the moderate ER under nutrient starvation. Overall, our study has shown that the slow ER becomes a major bottleneck for synthesizing stress-defensive protein in *E. coli* during oxidative stress, which has not observed in other stress conditions. Given such a severe physiological burden of translational elongation stress under oxidative stress, an intriguing question is why *E. coli* cells evolve such tRNA-degradation response. A possible explanation may lie in the concern of translational accuracy (52,53). It has been known that oxidative stress could cause mild protein mistranslation (54). In this case, high tRNA pools and the resultant high translational elongation rate may further exacerbate the translation error problem and compromise cell viability under oxidative stress (52).

At 0.1–5 mM H_2_O_2_, *E. coli* cells could resume growth after a certain lag time. It should be noted that although the cellular tRNA pools and translation capacity gradually recover during the lag time, cell growth does not gradually recover. Instead, the growth recovery occurs rapidly at the end of lag time. The origin is likely to be that: bacterial cells are very sensitive to the treatment of H_2_O_2_. H_2_O_2_ causes cell growth arrest through inhibiting the activity of related metabolic enzymes in TCA cycle and some key biosynthesis pathways (16,17). As little as micromoles (∼μM) levels of H_2_O_2_ are enough to inhibit cell growth (16,55). Therefore, although translation capacity is recovering with the removal of H_2_O_2_ during the lag time, cell might not be able to resume growth until most H_2_O_2_ has been removed. On the other hand, it is intriguing that the *E. coli* cells can completely resume its normal growth rate after the lag time. This observation indicates that the concentrations of essential components such as ribosome, RNAP and tRNA charging enzymes are maintained during H_2_O_2_ shock and are ready for use immediately after the lag time. For example, the cellular ribosome content (indicated by RNA/protein ratio) of *E. coli* cells does not change during H_2_O_2_ shock (blue asterisk in Supplementary Figure S10).

In summary, our finding elucidate that the tRNA-limited translational elongation process is the key physiological bottleneck that limits the bacterial survival during oxidative stress. The oxidative-induced growth arrest and cellular defense response is also a conserved phenomenon in yeast cells and mammalian cells (11,14,15). Furthermore, substantial tRNA degradation under oxidative stress has also been reported in yeast cells and mammalian cells (40–42,56). Therefore, the notion of our study might be applicable to eukaryotic cells as well, which deserves to be explored in the future for better understanding of the ROS physiology of eukaryotes.

## Supplementary Material

gkz467_Supplemental_Files
