# Supplementary material for: Maintenance of translational elongation rate underlies the survival of Escherichia coli during oxidative stress
Source: Nucleic Acids Res. 2019 May 27;47(14):7592–604. doi: 10.1093/nar/gkz467 (PMC6698664; doi:10.1093/nar/gkz467)
Supplement: gkz467_Supplemental_Files [file gkz467_supplemental_files.pdf]

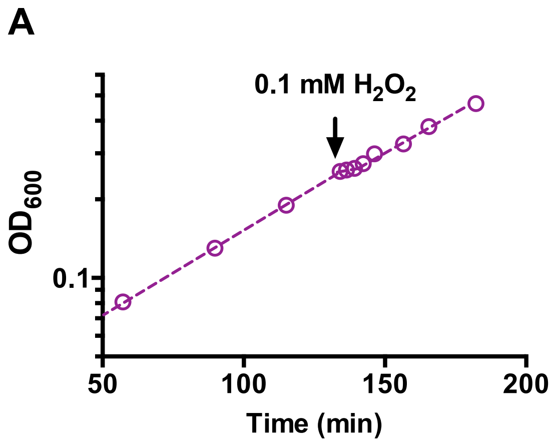

Figure S1 The growth curve of *E. coli* cells being subjected to the treatment of 0.1 mM and 0.25 mM hydrogen peroxide (H_2O_2). The lag times of *E. coli* cells at 0.1 mM and 0.25 mM H_2O_2 are 5 min and 9 min, respectively.

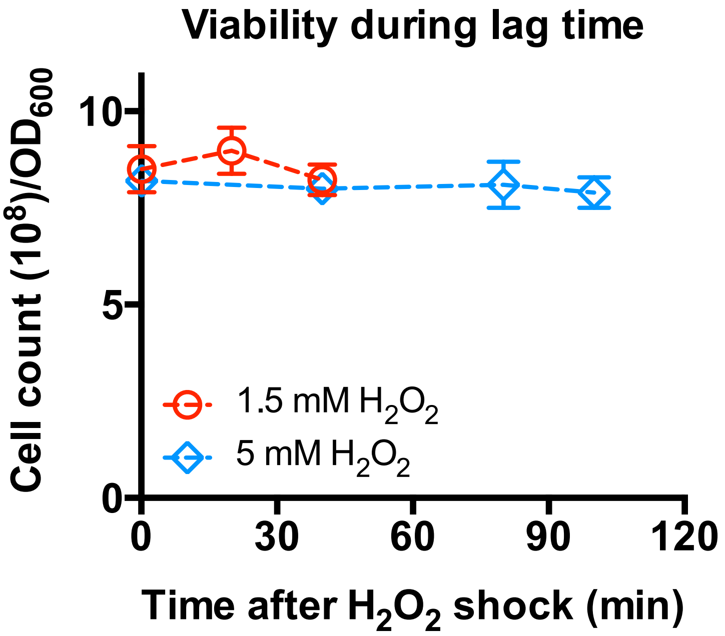

Figure S2 Cell viability of *E. coli* during the lag time of H_2O_2 treatment. The lag times of *E. coli* cells subjecting to 1.5 mM H_2O_2 and 5 mM H_2O_2 are 38 min and 90 min, respectively. The cell viability of 1.5 mM H_2O_2 was measured by plating at 0 min, 20 min and 40 min after addition of H_2O_2. The cell viability of 5 mM H_2O_2 was measured by plating at 0 min, 40 min, 80 min and 100 min after addition of H_2O_2.

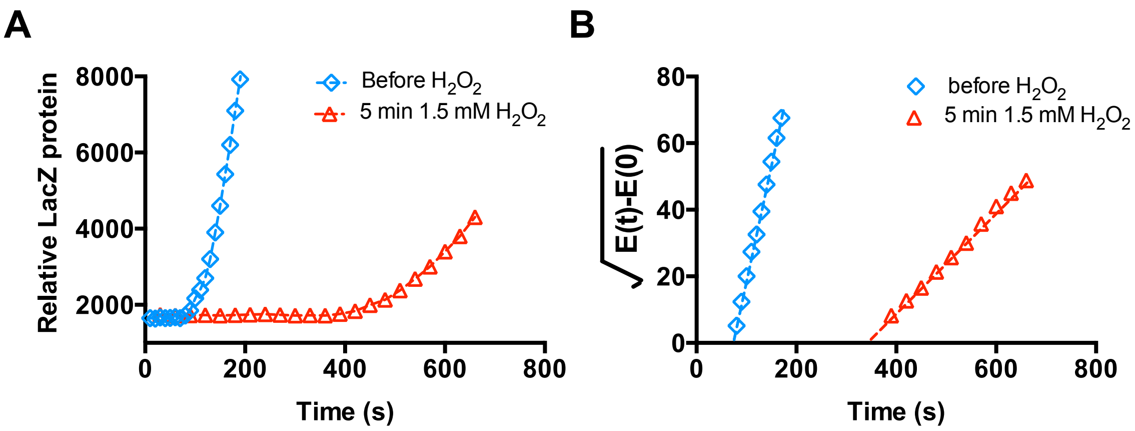

Figure S3 Measurement of translational elongation rate (ER) by LacZ induction assay. LacZ induction assay was used to determine the waiting time of the synthesis of the first LacZ protein after IPTG addition. It has been extensively used for the measurement of ER in Ref. 25 and Ref 26 of main text. IPTG was added to induce the expression of LacZ protein. **(A)** The LacZ induction curve of *E. coli* cells in glucose medium before H_2O_2 treatment and at 5 min after the addition of 1.5 mM H_2O_2. The LacZ activity of the culture was plotted against the induction time after IPTG addition. **(B)** The Schleif plot of the LacZ induction curve in panel A. The Schleif plot is used to deduce the translational time of the first newly synthesized LacZ molecule after the addition of IPTG. The square root of the newly synthesized LacZ ($\sqrt{E(t)-E(0)}$) was plotted against the induction time. The *E* (0) denotes the basal LacZ activity of the culture, and the *E* (t) denotes the LacZ activity at specific time points after addition of IPTG. During the initial several minutes, $\sqrt{E(t)-E(0)}$ is linear correlated with the induction time, and therefore the X-intercept of the linear line corresponds to the time needed for ribosome to translate a full-length LacZ molecule (T_{first}). From Panel B, the T_{first} was significantly larger upon H_2O_2 treatment, suggesting a much slower ER. ER equals to 1024/ (T_{first}-10), where 10-s denotes the time cost of initiation steps, including IPTG penetration into cells, LacI de-repression, RNA polymerase transcriptional initiation and ribosome translational initiation. The initiation time, being deduced by LacZα induction assay, was found to be constant at ~10 s at various growth conditions including nutrient limitation, antibiotic treatment, nutrient starvation and high osmolarity (Ref 25 and Ref 26 of main text). As deduced in Figure S4, the initiation time is still ~10 s at H_2O_2 shock. The T_{first} of *E. coli* cells before H_2O_2 shock and at 5 min after 1.5 mM H_2O_2 shock is 74 s and 344 s, corresponding to an ER of 16 aa/s and 3.1 aa/s, respectively.

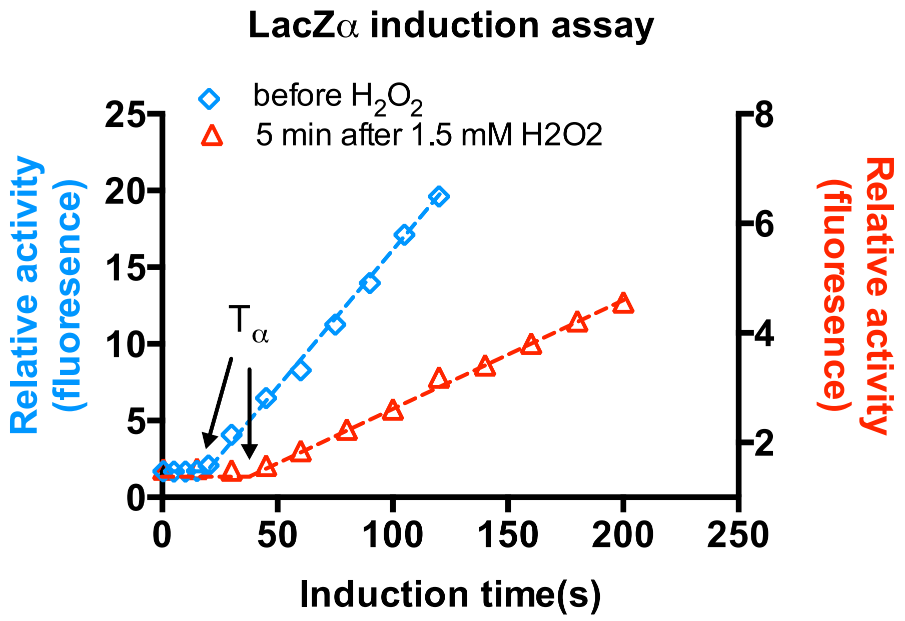

Figure S4 LacZα induction assay upon H_2O_2 treatment. The induction time of LacZ protein (shown in Figure S3) includes the time cost of several initiation steps such as IPTG penetration into cells, LacI de-repression, RNA polymerase transcriptional initiation and ribosome translational initiation. To accurately compute the elongation time, we need to estimate and subtract away the time cost of initiation steps. Towards this end, we performed a similar induction kinetics study for the LacZ alpha fragment (or LacZα, containing the N-terminal 1-90 aa of LacZ), as detailed in the supplementary text of Ref. 25-27. In those studies, the initiation time was found to be constant at ~10 s at various growth conditions including nutrient limitation, antibiotic treatment, nutrient starvation and high osmolarity. Here, we also performed the LacZα induction assay of *E. coli* at 5 min after the addition of 1.5 mM H_2O_2 (red triangles). *E. coli* cells were growing in glucose minimal medium. We first estimated the synthesis time of LacZα (T_α) by using flat line for the first few points and least square line-fit for the remaining points. The x-coordinates of the intersection points, T_α, are 16 s and 39 s respectively for *E. coli* cells before the H_2O_2 treatment and at 5 min after addition of 1.5 mM H_2O_2. The initiation time, $T_{init} = T_\alpha - 90/ER$, where the 90/ER is the elongation time need to synthesize the 90-aa LacZα fragment. As a first estimate of the elongation rate ER, we used ER = $934/(T_{first}-T_\alpha)$, where T_{first} is the time needed to synthesize of the first full-length LacZ molecular (Figure S3). T_{first}- T_α is the elongation time needed to translate the rest 934 residues of LacZ downstream of the LacZα part. Therefore, the T_{init} is 10.4 s and 9.6 s respectively for *E. coli* cells before H_2O_2 treatment and at 5 min after 1.5 mM H_2O_2 treatment. Therefore, the T_{init} is still ~10 s in the case of H_2O_2 treatment. ER equals to 1024/ (T_{first}-10).

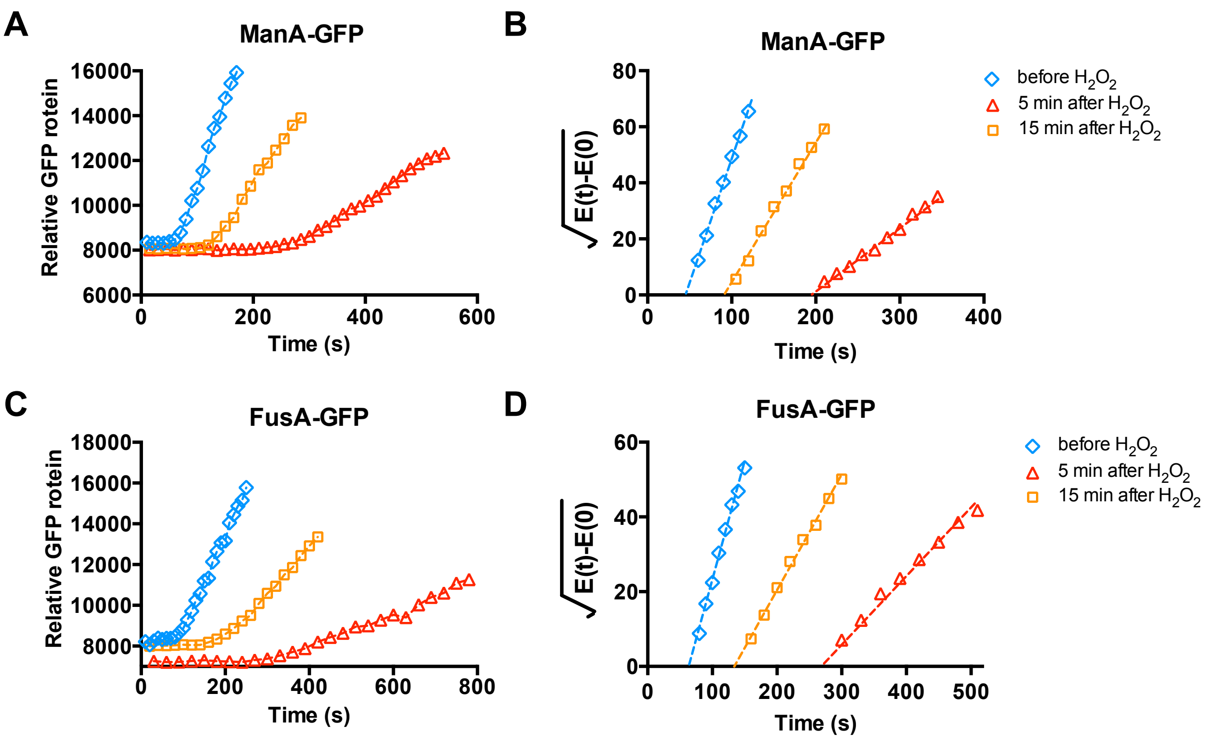

Figure S5 Induction assay of ManA-GFP and FusA-GFP protein upon 1.5 mM H_2O_2 treatment. Translational elongation rate was also measured for two GFP fusion proteins, ManA-GFP (length: 639 aa) and FusA-GFP (length: 952 aa) using similar induction assay as LacZ protein. These two gfp-fused genes were driven by the IPTG-inducible *Ptac* promoter under the control of *PlacIq-lacI* cassette (see methods part). Schelif plot was also used for measuring the T_{first} of each GFP protein.

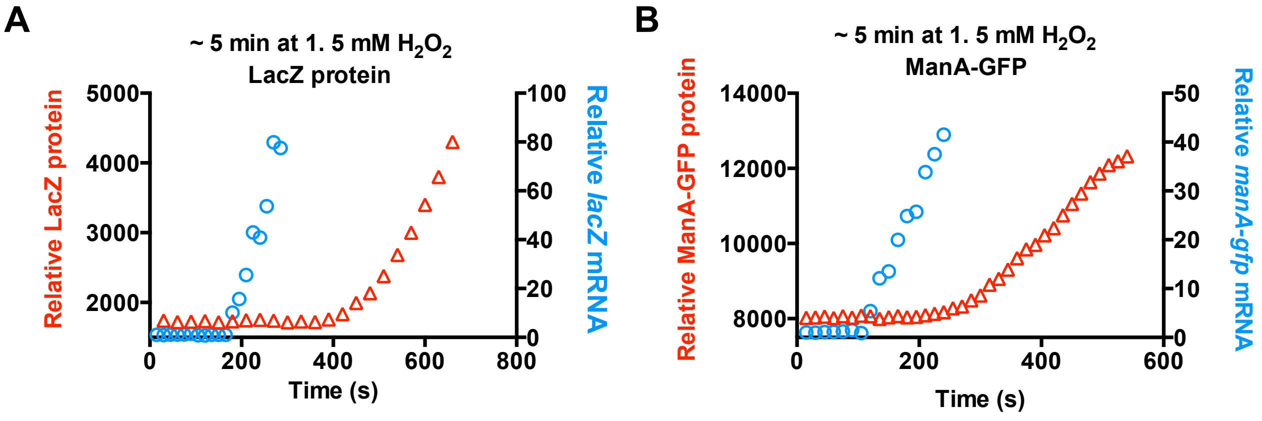

Figure S6 Induction assay of full-length *lacZ* mRNA and full-length *manA-gfp* mRNA at ~5 min after addition of 1.5 mM H_2O_2. (A) *lacZ* mRNA (blue circles); **(B)** *manA-gfp* mRNA (blue circles). The mRNA abundance was quantified by qRT-PCR. A pair of qPCR primers was used to detect the 3' end region of *lacZ* mRNA and *manA-gfp* mRNA, respectively. In this case, the qRT-PCR detects the synthesis kinetics of full-length mRNA. The induction curve of protein was also shown in red.

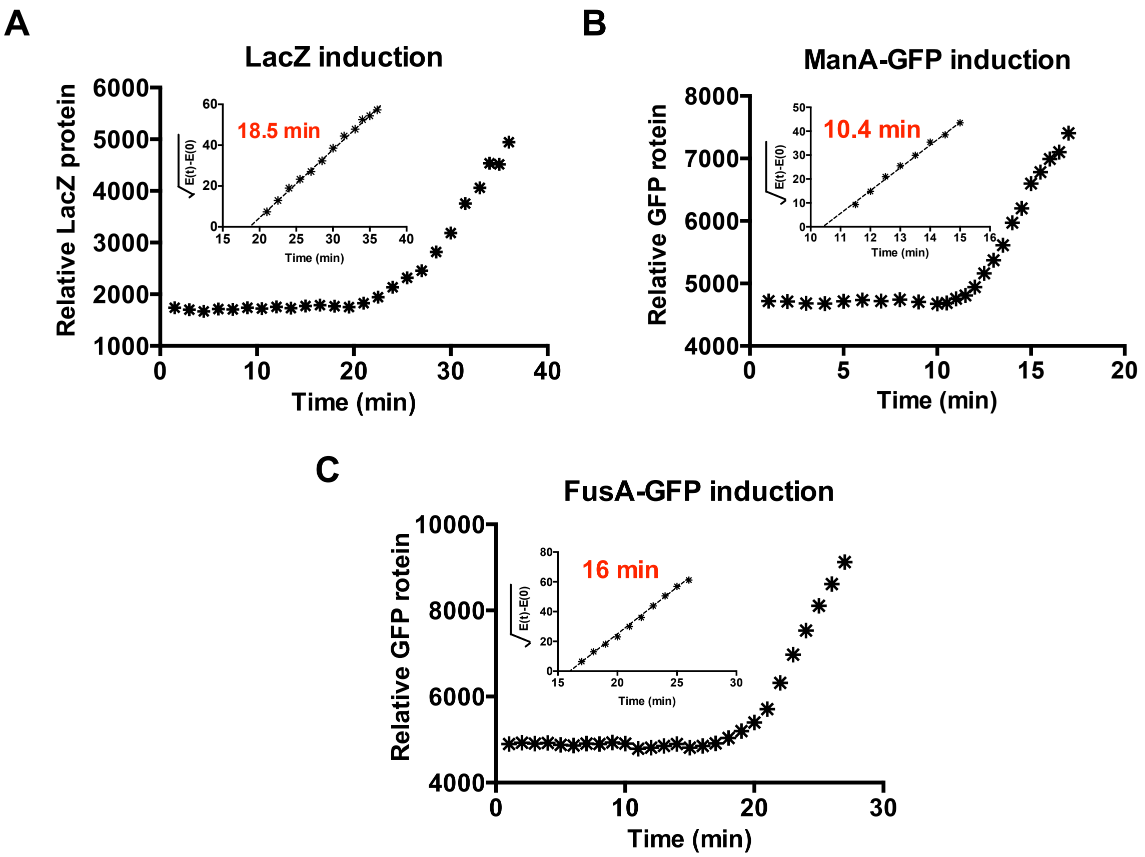

Figure S7 Induction curves of LacZ, ManA-GFP and FusA-GFP proteins of *E. coli* cells at 5 min after the addition of 5 mM H_2O_2. The insert of each panel shows the Schleif plot of each induction curve. The red number shows the time needed to synthesize the full-length protein.

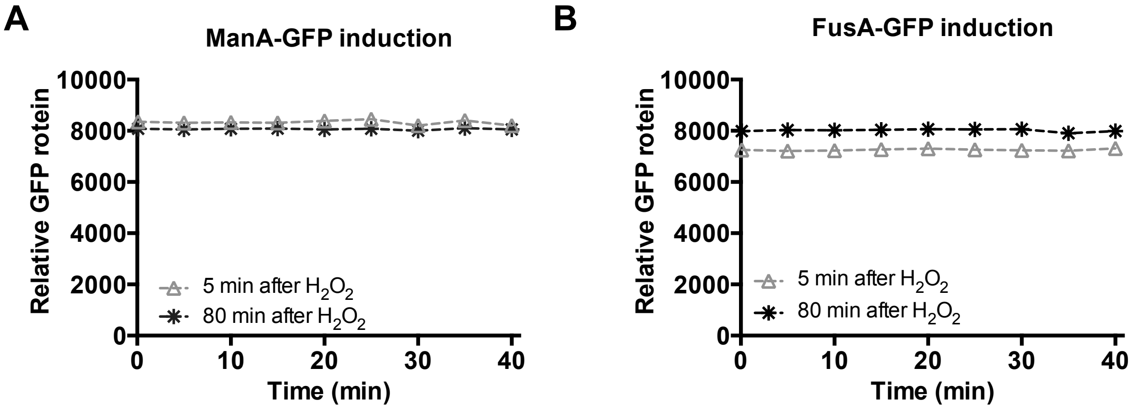

Figure S8 Induction curve of GFP fusion protein upon 6 mM H_2O_2 shock. The induction curves of ManA-GFP and FusA-GFP protein for *E. coli* cells growing in glucose minimal medium at two time points (5 min and 80 min) after the addition of 6 mM H_2O_2.

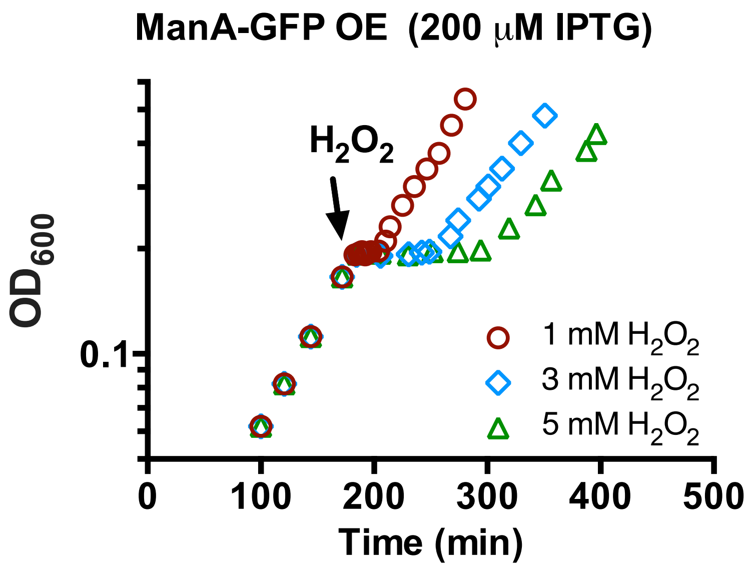

Figure S9 Growth curve of H_2O_2-treated *E. coli* cells upon overexpression of ManA-GFP protein. The FL174 strain harboring pFL-ManA-GFP vector was exponentially growing in glucose minimal medium supplemented with 200 μM IPTG.

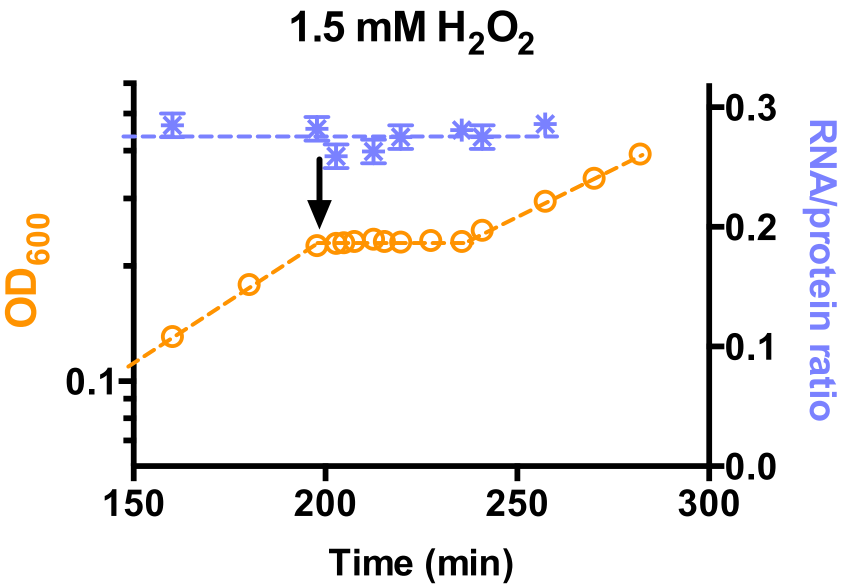

Figure S10 The cellular ribosome content (RNA/protein ratio) of *E. coli* subjecting to 1.5 mM H_2O_2 treatment. The RNA/protein ratio is shown together with the growth curve of *E. coli* cells subjecting to 1.5 mM H_2O_2 treatment. The growth curve is the same as shown in Figure 1A. The RNA/protein ratio is an accurate measurement of the cellular ribosome content as reviewed in Bremer and Dennis (Ref 28 of main text). In recent studies, RNA/protein ratio is further found to be proportional to ribosomal protein in many extreme conditions including extreme poor nutrient conditions, chloramphenicol inhibition and hyperosmotic stress (Ref 25 and Ref 26 of main text).
